# Integrated Transcriptomic and Physiological Analyses Reveal Disruption of Digestive Homeostasis Induced by Protease Inhibitors in *Anticarsia gemmatalis* Caterpillars

**DOI:** 10.1002/arch.70223

**Published:** 2026-10-04

**Authors:** Neilier Rodrigues da Silva Júnior, Eulálio Gutemberg Dias dos Santos, Daniel Guimarães Silva Paulo, Yaremis Meriño‐Cabrera, Ian de Paula Alves Pinto, Maria Julia Summy, Ian Lucas Batista Santos, Milena Godoi Lima, Maria Clara Neves Gomes Rodrigues, Rafael Junior de Andrade, José Eduardo Serrão, Elizabeth Pacheco Batista Fontes, Humberto Josué Oliveira Ramos, Maria Goreti de Almeida Oliveira

**Affiliations:** ^1^ Department of Biochemistry and Molecular Biology Laboratory of Enzymology and Biochemistry of Proteins and Peptides, Universidade Federal de Viçosa, UFV, BIOAGRO/INCT‐IPP Viçosa Brazil; ^2^ Department of General Biology Universidade Federal de Viçosa Viçosa Brazil; ^3^ Department of Biochemistry and Molecular Biology, Laboratory of Plant Molecular Biology Universidade Federal de Viçosa, UFV, BIOAGRO/INCT‐IPP Viçosa Brazil; ^4^ Núcleo de Análise de Biomoléculas, NuBioMol, Universidade Federal de Viçosa Viçosa Brazil

**Keywords:** digestive proteases, midgut transcriptome, oxidative stress, protease inhibitors

## Abstract

Protease inhibitors (PIs) are promising bioactive compounds for sustainable pest management because they interfere with the digestive processes of herbivorous insects. However, the physiological mechanisms underlying insect adaptation to natural and synthetic PIs remain poorly understood. Here, we investigated the midgut responses of *Anticarsia gemmatalis* Hübner (Lepidoptera: Erebidae) to the soybean Kunitz trypsin inhibitor (SKTI) and the synthetic tripeptide GORE‐2 using integrated transcriptomic, biochemical, and histological approaches, complemented by an exploratory metatranscriptomic analysis of putative gut‐associated microbial transcripts. RNA‐seq analysis identified 2542 unique differentially expressed genes (FDR‐adjusted *p* < 0.05, |log2FC | ≥ 1), including extensive modulation of digestive proteases and redox‐related pathways. SKTI induced a compensatory response characterized by the up‐regulation of serine proteases, antioxidant enzymes, and detoxification‐associated pathways. In contrast, GORE‐2 promoted broader repression of digestive proteases, including chymotrypsin‐ and trypsin‐related transcripts and matrix metalloproteinases, together with a distinct antioxidant response. Biochemical assays demonstrated increased CAT, GST, POX, and SOD activities under both treatments. Compared with SKTI, GORE‐2 induced lower POX, GST, and SOD activities, whereas CAT activity did not differ significantly between the two treatments. Histological analyses demonstrated notable epithelial disruption, vacuolization, and disorganization of the striated border in the larval midgut, with lesion scores being significantly elevated following GORE‐2 exposure. Exploratory analysis of putative gut‐associated microbial transcripts recovered from whole‐midgut RNA‐seq revealed taxonomically diverse microbial signals associated with carbon metabolism, proteolysis, and detoxification. However, no treatment‐associated differential expression was detected among the putative bacterial transcripts. These findings indicate that GORE‐2 induced broader disruption of digestive and protective midgut functions than SKTI, with weaker antioxidant enzyme induction and more pronounced histological injury, consistent with reduced physiological compensation in *A. gemmatalis* larvae. These results underscore the potential of synthetic peptide‐based protease inhibitors to interfere with adaptive digestive processes in pest insects of agricultural importance.

## Highlights

- Synthetic and plant protease inhibitors induce distinct midgut responses.
- SKTI triggers compensatory up‐regulation of digestive proteases.
- GORE‐2 suppresses digestive enzymes and weakens antioxidant defenses.
- GORE‐2 causes more severe epithelial disruption in the insect midgut.

## Graphical abstract

The peptides SKTI and GORE‐2 triggered distinct midgut responses in *Anticarsia gemmatalis* (Lepidoptera: Erebidae). SKTI promoted compensatory digestive and antioxidant responses, whereas GORE‐2 caused broader digestive protease repression, weaker antioxidant activation, and greater epithelial damage. Exploratory metatranscriptomic analysis detected putative gut‐associated microbial transcripts, with no treatment‐associated differential expression.

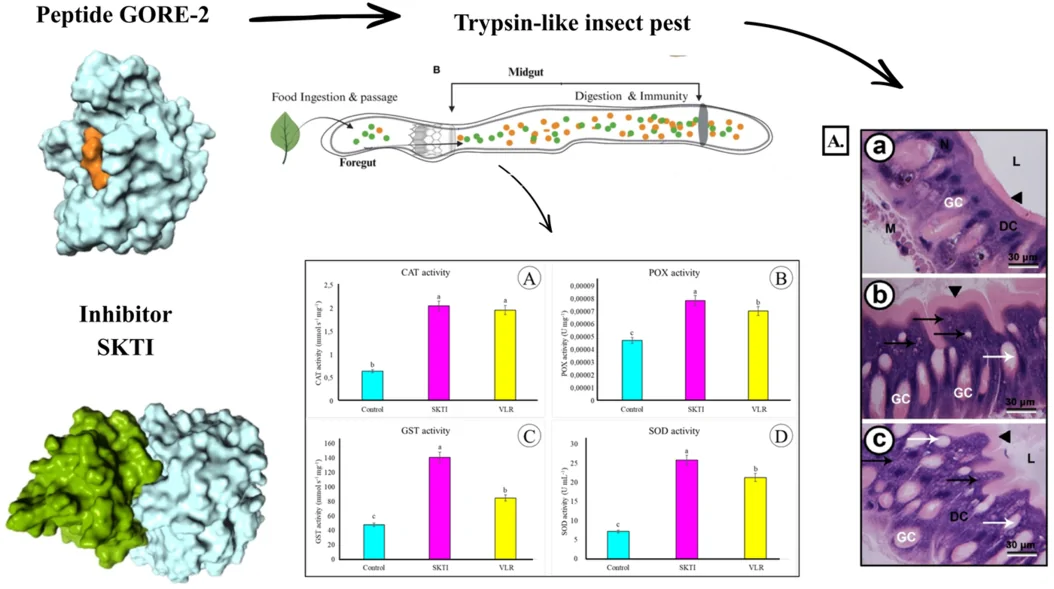

## 1 Introduction

*Anticarsia gemmatalis* Hübner (Lepidoptera: Erebidae), commonly known as the velvetbean caterpillar, is a major soybean pest responsible for severe defoliation and significant economic losses in Brazil and other soybean‐producing countries (de F. Bueno et al. 2026). Although the use of biological and organic insecticides has been effective in controlling this pest (Fiaz et al. 2018), integrated pest management (IPM) strategies often rely on synthetic chemical insecticides (Bueno et al. 2011) or transgenic soybean cultivars expressing *Bacillus thuringiensis* (Bt) toxins (Paula et al. 2014). However, the intensive use of pesticides has led to the emergence of resistant insect populations (Coelho et al. 2020; Horowitz et al. 2020) and caused collateral effects on non‐target organisms, soil, aquatic ecosystems, and human health (Campos et al. 2019; Sousa et al. 2020). These challenges underscore the need for alternative, sustainable pest‐control molecules that are selective, biodegradable, and environmentally safe (Moloi and Ngara 2023).

Protease inhibitors (PIs) represent a promising class of bioactive compounds for pest management because they are naturally occurring, non‐persistent in the environment, and selective toward target species. PIs are proteins or small peptides that interact with digestive proteases in the insect gut, reducing protein hydrolysis and nutrient assimilation (Pontual et al. 2012). This inhibition impairs larval development and reproductive success (de Almeida Barros et al. 2022, 2021; Schultz et al. 2024; Paulo, Schultz, et al. 2026; Paulo, Schneider, et al. 2026). Nevertheless, large proteinaceous PIs such as the soybean Kunitz trypsin inhibitor (SKTI) often contain cleavage sites susceptible to hydrolysis by insect proteases (Zhao et al. 2010; Singh et al. 2020) and many lepidopterans can express inhibitor‐insensitive isoforms (Coura et al. 2022; da Silva Júnior et al. 2020). To overcome these adaptive countermeasures, a rationally designed tripeptide, GORE‐2, was synthesized to selectively target the catalytic triad of *A. gemmatalis* gut trypsin‐like proteases and act as a potent, stable inhibitory molecule (de Almeida Barros et al. 2021). The effects of SKTI and GORE‐2 on larval survival and development were previously established by Barros et al. (de Almeida Barros et al. 2021), the present work specifically focused on elucidating the early molecular and physiological mechanisms associated with protease inhibitor exposure in the larval midgut.

The physiological consequences of PI ingestion extend beyond digestive inhibition. PIs can trigger systemic responses, including reprogramming of protease gene expression (Coura et al. 2022), activation of detoxification enzymes (Farahani et al. 2020), and modulation of redox homeostasis (Paulo, Meriño‐Cabrera, et al. 2026). Insects rely on an intricate antioxidant defense system to mitigate the harmful effects of reactive oxygen species (ROS), which, at controlled levels, play crucial roles in signaling and immunity (Lijun et al. 2005; Wang et al. 2006). Elevated ROS levels, however, can damage cellular components, eliciting the activation of antioxidant enzymes such as catalase (CAT), peroxidase (POX), glutathione S‐transferase (GST), and superoxide dismutase (SOD), which are recognized biomarkers of oxidative stress and insecticide resistance (Farahani et al. 2020; Jahan et al. 2008).

In this study, we investigated the molecular and physiological responses of *A. gemmatalis* larvae to two protease inhibitors with distinct structural origins: the natural soybean SKTI and the synthetic tripeptide GORE‐2. Using an integrative approach combining transcriptomic (RNA‐seq), biochemical, histological, and metatranscriptomic analyses, we evaluated how these inhibitors affect digestive protease expression, antioxidant and detoxification responses, intestinal integrity, and the potential contribution of the gut microbiome under PI exposure.

## 2 Materials and Methods

### 2.1 Insects and Preparation of the Artificial Diet Containing PIs

*A. gemmatalis* caterpillars were obtained from the Insects Laboratory of the Department of Biochemistry and Molecular Biology of the Universidade Federal de Viçosa (UFV), Viçosa, Minas Gerais, Brazil, and maintained at 25 ± 2°C, 70 ± 10% relative humidity, and 12 h photophase. After egg hatching, the caterpillars were fed on an artificial diet according to Greene et al (Greene et al. 1976). The fifth instar larvae were individually placed in cages 3 × 5 cm and fed ad libitum on a diet (control) and diets supplemented with sublethal PIs concentration: 0.12% (w/w) of serine‐protease soybean Kunitz trypsin inhibitor (SKTI; protein content 70%–90%, determined by the Biuret method; Sigma‐Aldrich, St. Louis, MO, USA) (treatment 1) and 0.12% (w/w) of GORE‐2 (95% purity; Aminotech, Brazil) (treatment 2), as described by Chougule et al (Chougule et al. 2008). After 24 h, the caterpillars were collected from each treatment and control. The intestines were removed and stored at ‐80°C. For downstream biochemical and transcriptomic analyses, three independent biological replicates were used per treatment. Each biological replicate consisted of a pool of five midguts collected from independently treated fifth‐instar larvae. Thus, each treatment comprised 15 larvae for these analyses.

The 0.12% (w/w) concentration was selected based on previous bioassays showing that SKTI and GORE‐2 significantly impair the survival and development of *A. gemmatalis* caterpillar (de Almeida Barros et al. 2021). Therefore, the present study employed a sublethal exposure design to investigate early molecular, biochemical, and histological responses associated with protease inhibitor ingestion, while minimizing extensive mortality during the 24 h experimental period. A 24 h exposure period was chosen to assess integrated sublethal responses to protease inhibitor ingestion, enabling the detection of transcriptional, enzymatic, and histological changes before the onset of extensive mortality or advanced systemic deterioration. This design provides a comparable snapshot of early physiological responses in the larval midgut under both treatments.

### 2.2 Histological Assessment and Semiquantitative Scoring

For histological observations, five *A. gemmatalis* caterpillars fed on PIs and controls were collected, dissected, and transferred to Zamboni's fixative solution for 12 h at 4°C (Fiaz et al. 2018). The midguts were dehydrated in a graded ethanol series (70%, 80%, 90%, and 95%) and embedded in historesin (Leica) following the manufacturer's instructions. Sections three μm thick were obtained with a rotation microtome, stained with hematoxylin (8 min) and eosin (30 s), and analyzed with a light microscope. Histological alterations were further assessed by semiquantitative scoring of epithelial lesions in blinded micrographs. Four parameters were evaluated: vacuolization, brush border disorganization, epithelial detachment, and cellular disruption.

### 2.3 Antioxidant and Detoxifying Enzyme Assay

Catalase activity (CAT) was determined according to Aebi (Aebi 1984). The reaction mixture contained 100 µL of sample in a total volume of 1.5 mL of 30 mM H2O2 and 50 mM phosphate buffer (pH 7.4). Absorbance readings were obtained at 240 nm in automatic mode, with one reading per second for 120 s (Molecular Devices, SpectraMax ABS Plus). Specific catalase activity values were expressed in mmol s‐1 mg protein‐1.

Peroxidase (POX) activity was determined according to the method of Shannon et al (Shannon et al. 1966). with minor modifications. The reaction was initiated by adding 0.1 mL of enzyme extract to 1.45 mL of 0.1 M sodium phosphate buffer (pH 6.5), 0.8 mM H_2_O_2_, and 5 mM Guaiacol. The absorbance was read at 470 nm for 120 s at 1 s intervals on the spectrophotometer (Molecular Devices, SpectraMax ABS Plus). The final enzyme activity was expressed in U per gram of protein. One enzymatic unit (U) was defined as a change in absorbance of 0.1 units per minute under test conditions.

Glutathione S‐transferase (GST) activity was determined according to the method of Dalton et al (Dalton et al. 2009)., using 1‐chloro‐2,4‐dinitrobenzene (CDNB) as the substrate. 50 µL of enzyme extract was added to the reaction mixture containing 50 µL of CDNB, 150 µL of 50 mM reduced glutathione (GSH), and 2.5 mL of 100 mM sodium phosphate buffer (pH 6.5). The reaction mixture was carefully homogenized, and the formation of the GSH‐CDNB complex was monitored using a spectrophotometer (Molecular Devices, SpectraMax ABS Plus) at 340 nm for 120 s, with automatic readings every second. The activity was determined using the molar extinction coefficient ε = 9.6 mM^−1^ cm^−1^.

Superoxide dismutase (SOD) activity was determined using a commercial colorimetric kit (Sigma‐Aldrich, St. Louis, MO, USA), following the manufacturer's recommendations. For each treatment, three independent biological replicates were analyzed, with each biological replicate consisting of a pool of five larval midguts. Enzymatic measurements were performed in technical triplicate for each biological sample. Technical readings were averaged before statistical analysis and were not treated as independent observations.

### 2.4 Protein Extraction From the Caterpillar Intestines

For each treatment, three independent biological replicates were prepared, with each replicate consisting of a pool of five midguts. Each pool was transferred to 1 mL of 10−3 M hydrochloric acid solution at 4°C and homogenized using a TissueLyser II (Qiagen). The extracts were centrifuged at 10,000 × g for 30 min at 4°C, the pellets were discarded, and the resulting supernatants containing the soluble protein fraction were stored at −20°C until enzymatic analysis.

### 2.5 RNA Extraction and Sequencing

For each experimental condition (control, SKTI, and GORE‐2), three independent biological replicates were used for RNA‐seq. Each biological replicate consisted of a pool of five midguts collected from independently treated fifth‐instar larvae, resulting in a total of nine biological samples. Total RNA was independently extracted from each biological replicate using TRIzol reagent (Invitrogen), according to the manufacturer's instructions. DNA contamination was removed using RNase‐free DNase I (New England Biolabs). RNA purity and concentration were assessed using a NanoDrop spectrophotometer (Thermo Scientific) and a Qubit 2.0 Fluorometer (Life Technologies), respectively. RNA integrity was verified using the RNA Nano 6000 Assay Kit on an Agilent Bioanalyzer 2100 system (Agilent Technologies). One cDNA library was independently constructed from each biological replicate, resulting in nine independent sequencing libraries. Total RNA from each treatment was isolated from the midgut of A. gemmatalis using TRIzol reagent (Invitrogen) according to the manufacturer's instructions. DNA contamination was eliminated from the RNA samples using RNase‐free DNase I (New England Biolabs). The purity and quantification of total RNA were assessed using a NanoDrop spectrophotometer (Thermo Scientific) and a Qubit 2.0 Fluorometer (Life Technologies). RNA integrity was verified using the RNA Nano 6000 Assay Kit on the Agilent Bioanalyzer 2100 system (Agilent Technologies). High‐quality total RNA isolated from independent biological replicates of each *A. gemmatalis* population was used to construct cDNA libraries.

Transcriptome libraries were generated using the NEBNext Ultra RNA Library Prep Kit for Illumina (NEB), following the manufacturer's recommendations. After independent library preparation, the nine libraries were pooled only for multiplexed sequencing and sequenced by BGI Genomics (Hong Kong) on the DNBseq platform. No biological RNA samples were pooled before library construction. Data cleaning and filtering were performed by BGI Genomics using SOAPnuke version 2.1.4 (Chen et al. 2018), which included adapter removal and the discarding of reads in which 50% of bases had a Phred score below 20.

### 2.6 Quality Analysis and Assembly

The quality of the sequencing data was assessed using reports generated by FastQC version 0.11.7 (https://github.com/s-andrews/FastQC), and these reports were aggregated with MultiQC version 1.12 (https://github.com/ewels/MultiQC) (Ewels et al. 2016). Reads were processed to remove adapter sequences from the libraries using the “auto‐detect” configuration in TrimGalore version 0.6.7 (https://github.com/FelixKrueger/TrimGalore). Subsequently, sequences were trimmed and filtered using Trimmomatic software version 0.39 (https://github.com/usadellab/Trimmomatic) (Bolger et al. 2014) with the following parameters: HEADCROP:15; LEADING:3; TRAILING:3; SLIDINGWINDOW:4:20; MINLEN:50. The trimmed sequences underwent processing using Rcorrector software version 1.0.4 (https://github.com/mourisl/Rcorrector) (Song and Florea 2015), employing default settings and applying a k‐mer‐based sequence correction method.

*Ab initio* transcriptome assembly was performed using Trinity software version 2.8.5 (https://github.com/trinityrnaseq/trinityrnaseq) (Grabherr et al. 2011), with default settings and transcripts filtered to a minimum size of 500 nucleotides. Descriptive statistics of the assembly were obtained using the “TrinityStats.pl” script. The assembly quality was also evaluated by aligning reads to transcript sequences using Bowtie2 v2.2.6 (https://github.com/BenLangmead/bowtie2), with the default settings “–end‐to‐end” and “–sensitive.”

Following the assembly, transcripts were clustered into sets of non‐redundant Unigenes using the cd‐hit‐est tool from CD‐HIT software version 4.8.1 (http://weizhong-lab.ucsd.edu/cd-hit/) (Fu et al. 2012), with a minimum identity threshold of 98% for sequence clustering.

### 2.7 Prediction of ORFs

The Unigene sequences were analyzed to identify open reading frames (ORFs) using TransDecoder.LongOrfs tool version 5.5.0 (https://github.com/TransDecoder/TransDecoder/wiki) (Haas et al. 2013), considering only ORFs with a minimum size of 300 nucleotides.

The protein sequences encoded by the predicted ORFs were compared with proteins available in the UniProt Knowledgebase database (UniProtKB, http://www.uniprot.org) (UniProt Consortium 2013) using the BLASTp tool of BLAST version 2.11.0 (https://ftp.ncbi.nlm.nih.gov/blast/executables/blast+/LATEST/). Protein sequences were also compared with Hidden Markov Models (HMMs) of domain families from the Pfam database (http://pfam.xfam.org/) (Finn et al. 2016) using HMMER version 3.1b2 (https://github.com/EddyRivasLab/hmmer) (Finn et al. 2016). Alignments with an E‐value ≤ 1×10‐10 were considered significant. BLASTp alignments were also evaluated for sequence similarity, selecting those with a product coverage x similarity ≥ 40%.

### 2.8 Metatranscriptomic Analysis

Bacterial open reading frames (ORFs) were predicted from the translated unigenes using Prodigal software, version 2.6.3 (https://github.com/hyattpd/Prodigal). The analysis was performed using the ‐p meta mode, suitable for the metatranscriptomic nature of the samples. The predicted proteins were compared against the UniProtKB/Swiss‐Prot database V.2025_03 for bacterial taxonomy (https://www.uniprot.org/taxonomy/2).

Alignments were performed using BLASTx from BLAST+ version ≥ 2.6, with an e‐value threshold of ≤ 1e‐10, which translates the nucleotide queries into all six reading frames before aligning them against the protein database. The results were then filtered using a minimum criterion of ≥ 30% identity and ≥ 30% coverage, selecting the best hit for each predicted ORF. After the annotation analysis, a specific filtering step was performed to identify proteins annotated as enzymes in EC 3.4, corresponding to bacterial proteases/peptidases. This selection also followed the minimum thresholds of 30% identity and coverage. The microbial signal was recovered from whole‐midgut RNA‐seq libraries dominated by host transcripts, and the microbial data are therefore reported as an exploratory survey of putative gut‐associated microbial transcripts

Taxonomic analysis of the protein sequences was performed using Kaiju version 1.10.1 (https://github.com/bioinformatics-centre/kaiju). The tool was executed in greedy mode using the RefSeq database (https://www.ncbi.nlm.nih.gov/refseq/) to assign taxonomy to the annotated sequences and identify potential bacterial components or contaminants associated with the intestinal content.

### 2.9 Analysis of Differentially Expressed Transcripts

Transcript abundance was quantified using Kallisto software version 0.44.0 (https://github.com/pachterlab/kallisto) (Bray et al. 2016), which performs pseudo‐alignment of reads against the assembled Trinity transcript sequences. Differential expression was subsequently analyzed at the transcript (isoform) level, using the transcript identifiers reported.

Differential expression analysis was conducted independently for each treatment relative to the control diet (SKTI vs. control and GORE‐2 vs. control), with the DESeq. 2 package version 3.15 (https://bioconductor.org/packages/release/bioc/html/DESeq.2.html). This analysis aimed to quantify the number of aligned sequences per transcript using Kallisto data. The applied parameters were adjusted with a *p*‐value < 0.05 and ∣| log2FC |> 1.

Genes identified as differentially expressed were analyzed using BLAST2GO (http://www.blast2go.de) (Conesa et al. 2005) performing automated annotation against NCBI's arthropod nr database with an E‐value of 1 × 10^‐10^. The enrichment of metabolic pathways associated with the differentially expressed genes was assessed using the KOBAS tool (http://kobas.cbi.pku.edu.cn/) (Xie et al. 2011) with reference to the genomes of *Helicoverpa armigera* and *Bombyx mori*, using a hypergeometric statistical test.

### 2.10 Functional Annotation

The functional annotation of the Unigenes sequences was performed using the TRAPID tool version 2.0 (Van Bel et al. 2013), selecting the EggNOG database version 4.5.1 and applying an E‐value ≤ 1 × 10^−10^ for gene family attribution, functional domain identification, and gene ontology (GO) term attribution. Unigenes identified as differentially expressed were also annotated using the BLAST2GO software (https://github.com/blast2go-apps) (Conesa et al. 2005), performing an automatic annotation against Arthropoda protein sequences (NCBI Taxonomy ID 6656) available in the NCBI RefSeq Non‐Redundant proteins (nr) database, and considering an E‐value ≤ 1 × 10^−10^ to select significant alignments. The metabolic pathway enrichment of differentially expressed Unigenes was evaluated using the KOBAS tool (https://github.com/xmao/kobas) (Xie et al. 2011) with the hypergeometric test (adjusted *p*‐value < 0.05) and using the genome annotations of *Helicoverpa armigera* and *Bombyx mori* as references.

### 2.11 Statistical Analysis

Statistical analyses were performed using R software version 4.5.2. Independent biological replicates were considered the experimental units in all biochemical analyses. For CAT, POX, GST, and SOD activities, three independent biological replicates per treatment were analyzed, each consisting of a pool of five larval midguts. Enzymatic measurements were performed in technical triplicate, and the technical readings were averaged before statistical analysis. Normality of residuals and homogeneity of variances were evaluated using the Shapiro–Wilk and Levene tests, respectively. Treatment effects were assessed using one‐way ANOVA followed by Tukey's HSD multiple‐comparison test when significant differences were detected. Statistical significance was set at *p* < 0.05. Histological lesion scores were analyzed using the mean score obtained for each larva as the experimental unit.

## 3 Results

### 3.1 Histological Assessment

Exposure of *A. gemmatalis* larvae to protease inhibitors induced clear histological alterations in the midgut epithelium (Figure 1). In the control group, the intestinal epithelium showed preserved morphology, characterized by a monolayer of digestive cells and goblet cells with well‐defined striated borders, homogeneous cytoplasm, and nuclei with decondensed chromatin (Figure 1a). In larvae fed with SKTI for 24 h, the midgut epithelium exhibited evident cytoplasmic vacuolization and disorganization of the striated border (Figure 1b). These alterations were more pronounced in GORE‐2‐fed larvae, which showed intense vacuolization and marked disruption of the striated border (Figure 1c).

**FIGURE 1:**
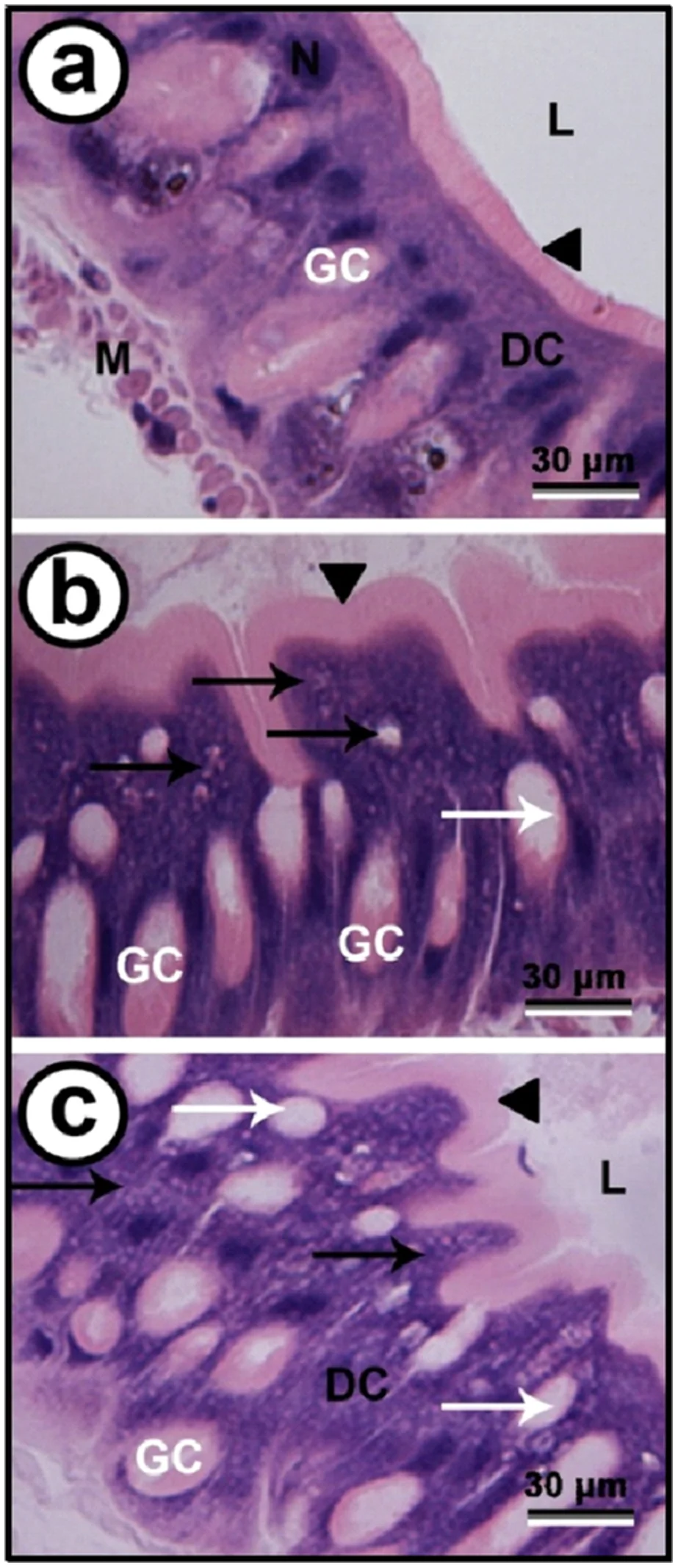
Histological alterations in the midgut epithelium of *Anticarsia gemmatalis* larvae after exposure to SKTI and GORE‐2. Representative light micrographs of the larval midgut epithelium. (a) Control group showing preserved epithelial morphology, with digestive cells (DC), goblet cells (GC), nuclei (N), muscle layers (M), lumen (L), and a well‐defined striated border (arrowhead). (b) SKTI‐fed larvae showing cytoplasmic vacuolization in digestive cells (black arrows) and disorganization of the striated border in goblet cells (white arrow). (c) GORE‐2‐fed larvae showing pronounced cytoplasmic vacuolization in digestive cells (black arrows) and disruption of the striated border in goblet cells (white arrows). Scale bars = 30 µm.

### 3.2 Antioxidant Enzyme Activity

Exposure of *A. gemmatalis* larvae to sublethal concentrations of protease inhibitors (PIs) for 24 h triggered marked biochemical responses consistent with the activation of oxidant defenses (Figure 2). Catalase (CAT) activity in the midgut increased approximately fourfold relative to the control in both PI treatments (Figure 2A), demonstrating a generalized activation of hydrogen peroxide detoxification pathways. No significant difference was observed between the SKTI‐ and GORE‐2–treated groups, suggesting comparable CAT induction under both treatments.

**FIGURE 2:**
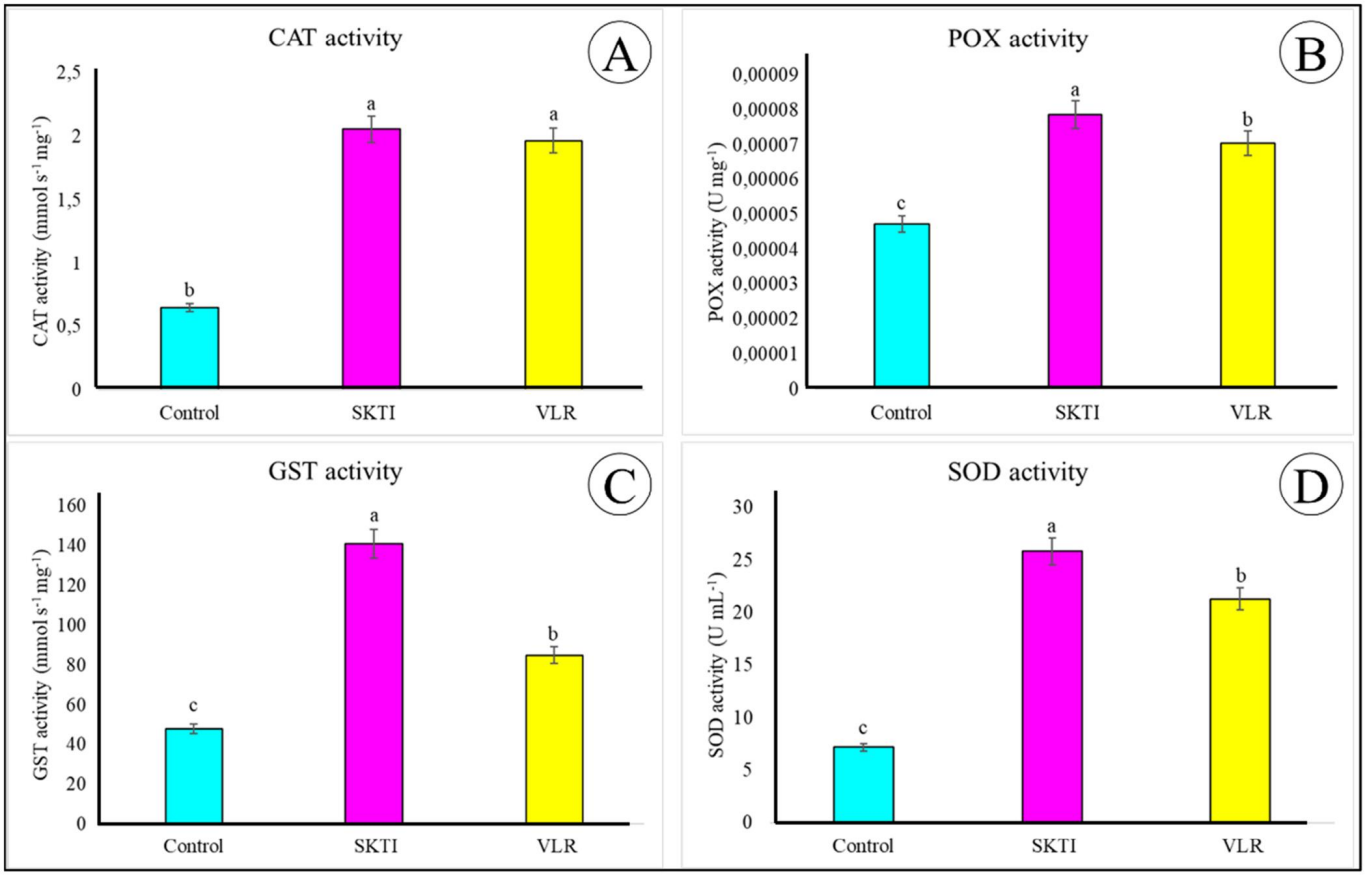
Effects of *A. gemmatalis* exposure to the protease inhibitors SKTI and GORE‐2 on the activities of four antioxidant and detoxifying enzymes: (A) catalase (CAT), (B) peroxidase (POX), (C) glutathione S‐transferase (GST), and (D) superoxide dismutase (SOD). Different letters above the bars indicate statistically significant differences between treatments (*p* < 0.05).

Peroxidase (POX) activity also rose significantly in response to both inhibitors (Figure 2B). However, the increase was more pronounced in SKTI‐fed larvae, with POX activity nearly 1.6‐fold higher than in the GORE‐2 group, reflecting a stronger peroxidative response to the proteinaceous inhibitor.

Similarly, glutathione S‐transferase (GST) activity was markedly enhanced (Figure 2C). SKTI feeding induced a 2.9‐fold increase relative to the control, while GORE‐2 promoted a 1.7‐fold increase. A significant inter‐treatment difference was observed, with GST activity being ~40% lower in GORE‐2–fed larvae than in those exposed to SKTI, indicating distinct impacts of each PI on phase‐II detoxification capacity.

For superoxide dismutase (SOD), both inhibitors stimulated strong enzymatic activation (Figure 2D). SKTI‐fed larvae exhibited a 3.5‐fold increase in SOD activity, while GORE‐2 resulted in a 2.9‐fold elevation relative to the control.

### 3.3 Data Analysis, Sequencing, and Transcriptome Assembly

The *A. gemmatalis* sequencing of midgut libraries generated 46–48 million 100‐nucleotide reads with an average GC content of 48%. After removing the adapters and trimming the reads, the sequencing data contained 43–44 million reads, with sizes ranging from 50 to 85 nt.

The reference transcriptome assembly identified 51,825 transcripts. The mean length of the transcripts obtained was 2243.6 nt, and the N50 was 3246 nt (Table S1). The transcriptome was validated by read mapping: 85.69% of the reads aligned to the assembly transcripts (20.33% aligned uniquely and 65.36% had multiple alignments), and 14.31% did not align. Furthermore, 20.33% aligned uniquely, and 65.36% had multiple alignments. The transcripts were grouped into 42,372 Unigenes, which have an average size of 1467 nt and an N50 of 2894 nt.

### 3.4 Functional Annotation

A total of 42,372 Unigenes were identified as gene candidates, and 29,565 complete open reading frames (ORFs) were located. Of these, 21,667 proteins were identified with complete ORFs and annotated for similarity to the UniProt database, resulting in a 50% annotation rate. The species *Bombyx mori* and *Danaus plexippus* accounted for approximately 70% of the best hits in the EggNog database (Figure 3). Additionally, 13,731 Gene Ontology (GO) terms were assigned based on Gene Ontology terms, and 13,223 enzyme KO terms were assigned for the KEGG database. The most frequent enzymes among the transcripts belonged to the classes of hydrolases (E.C. 3), transferases (E.C. 2), and oxidoreductases (E.C. 1). The trypsin‐like enzymes (KO1312) had 145 occurrences, being the most frequently encountered code (Figure 4). A total of 32,673 genes were annotated with protein domains according to the Pfam database.

**FIGURE 3:**
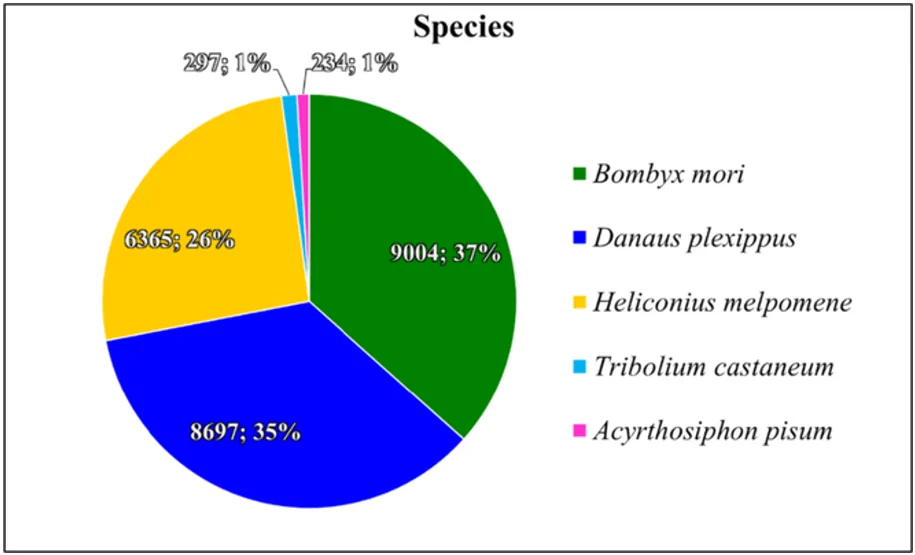
Distribution of functional annotations by sequence similarity across different species, calculated using the eggNOG database through the TRAPID tool.

**FIGURE 4:**
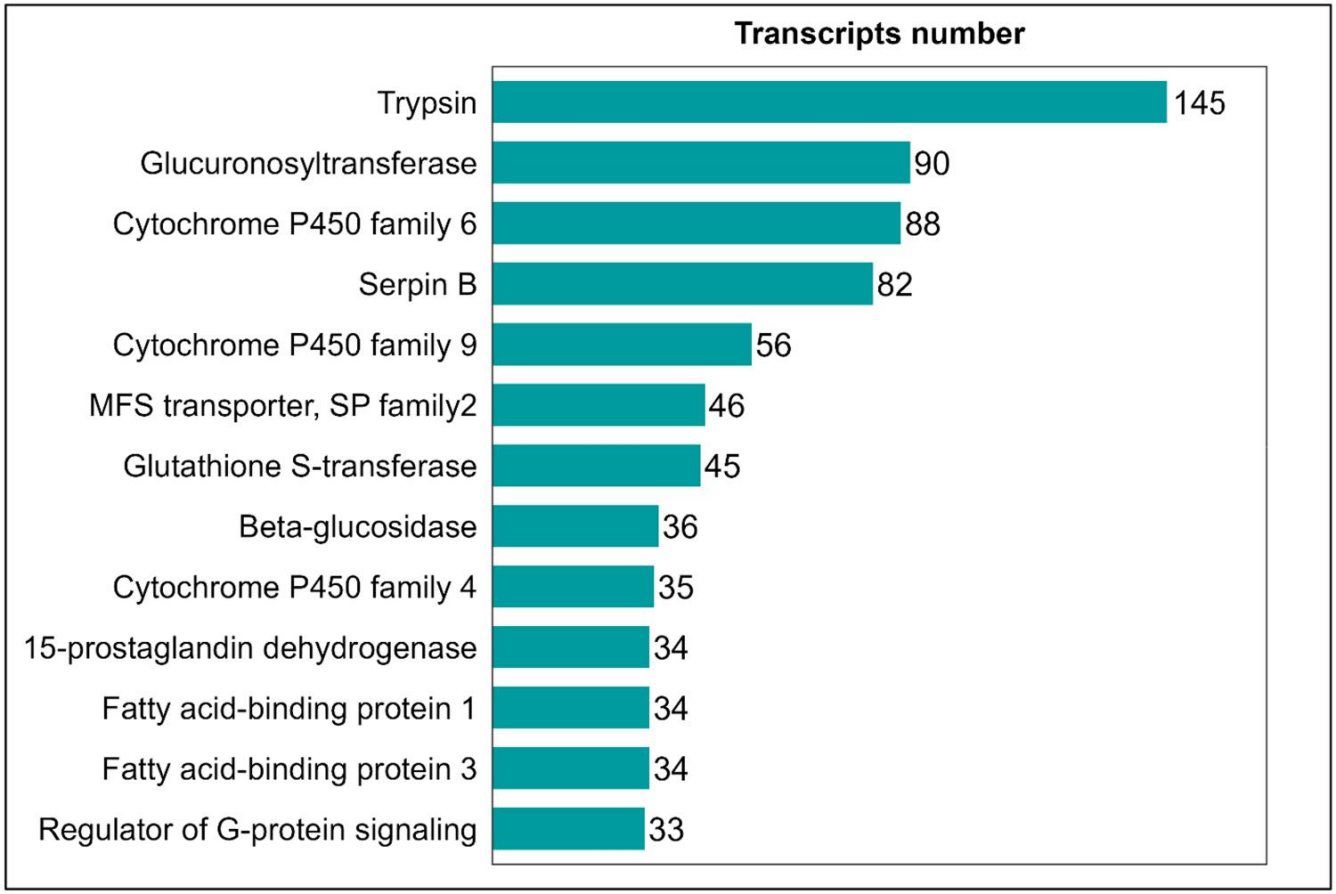
Most frequent protein domains identified in the *Anticarsia gemmatalis* transcriptome, annotated based on KEGG pathway analysis.

### 3.5 Analysis of Differentially Expressed Genes (DEGs)

A total of 2542 unique differentially expressed genes (DEGs) were detected across treatments. Among caterpillars fed the SKTI‐supplemented diet, 1507 genes exhibited altered expression, comprising 914 up‐regulated and 593 down‐regulated transcripts. In contrast, larvae treated with the tripeptide GORE‐2 showed 1736 DEGs, of which 984 were up‐regulated and 752 down‐regulated. Notably, 701 DEGs were shared between both treatments (556 concordantly up‐regulated, 140 concordantly down‐regulated, and five regulated in opposite directions), suggesting a partially overlapping transcriptional response possibly related to common stress or defense pathways. Unique DEGs in each treatment likely reflect compound‐specific effects on midgut physiology, digestive enzyme expression, and detoxification processes (Figure 5).

**FIGURE 5:**
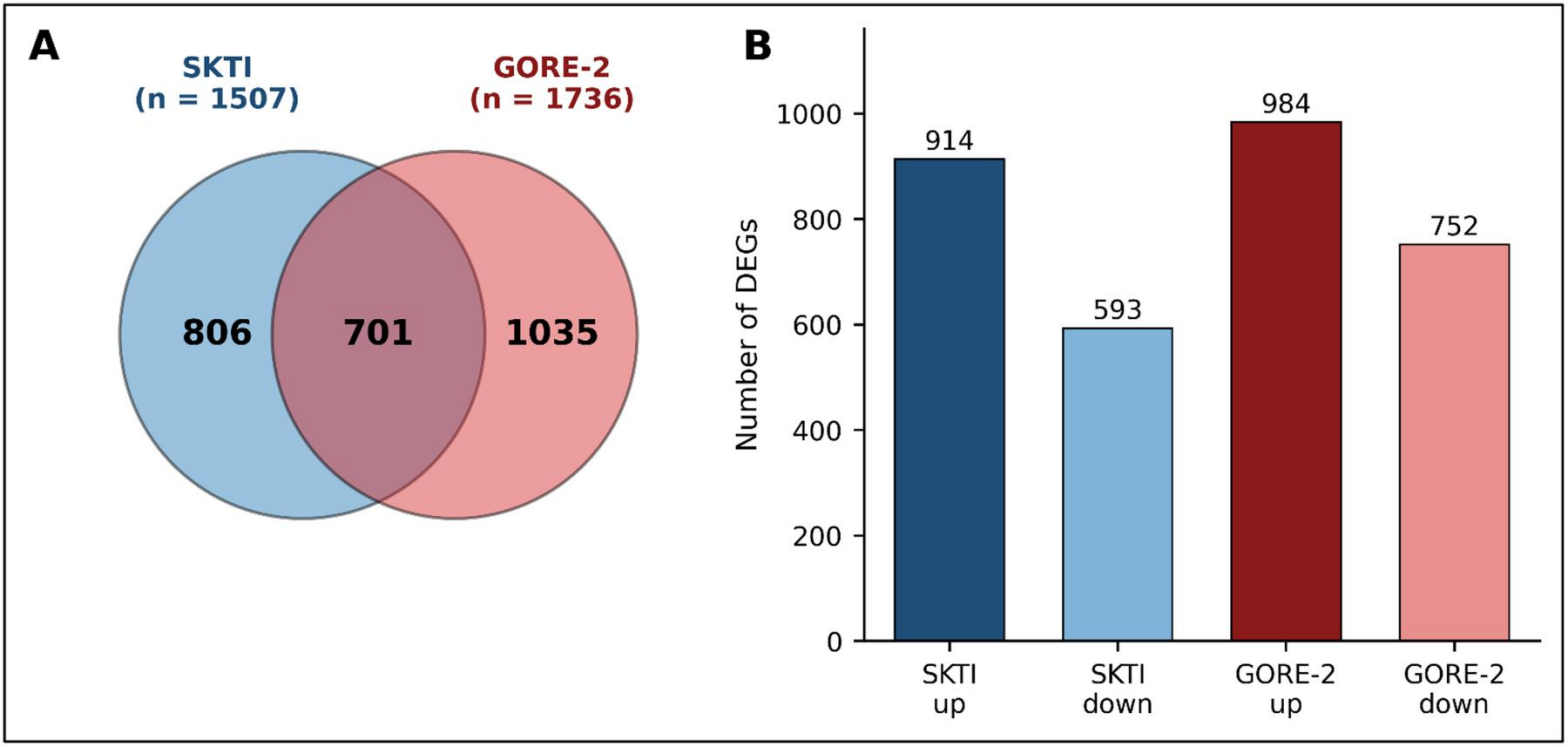
Venn diagram illustrating the Distribution of differentially expressed genes (DEGs) in the midgut of *A. gemmatalis* larvae after 24 h of exposure to SKTI or GORE‐2 (FDR‐adjusted *p* < 0.05, |log2FC | ≥ 1). (A) Venn diagram of the two contrasts against the control diet: 806 DEGs were exclusive to SKTI, 1,035 were exclusive to GORE‐2, and 701 were shared, totaling 2542 unique DEGs. (B) Number of up‐ and down‐regulated DEGs in each treatment (SKTI: 914 up, 593 down; GORE‐2: 984 up, 752 down).

The analysis of DEGs revealed 75 differentially expressed protease‐encoding transcripts, corresponding to 60 unique transcripts (Table 1), with 24 up‐regulated and 8 down‐regulated by SKTI. Conversely, caterpillars treated with GORE‐2 exhibited increased expression of 19 proteases and decreased expression of 24 proteases (Figure 6). Fifteen protease transcripts responded to both inhibitors, always in the same direction (11 up‐regulated and 4 down‐regulated in both treatments).

**TABLE 1:** Differentially expressed protease‐encoding transcripts in the *Anticarsia gemmatalis* midgut after 24 h of exposure to SKTI or GORE‐2 (DESeq. 2, Benjamini‐Hochberg FDR‐adjusted *p* < 0.01 and |log2FC | ≥ 1)

Contrast	Transcript ID	UniProt	Annotation	baseMean	log2FC	*p*‐value	FDR‐adj. p
SKTI versus control (up)	TRINITY_DN2479_c0_g1_i3	A0A2A4J4T7	Peptidase_M14 domain‐containing protein	399.3	23.95	1.95e‐10	4.68e‐08
SKTI versus control (up)	TRINITY_DN1362_c1_g1_i14	A0A6J1WCK9	cysteine proteinase B‐like	278.2	11.39	1.09e‐03	3.02e‐02
SKTI versus control (up)	TRINITY_DN2218_c0_g1_i21	Q8IS84	Serine protease inhibitor serpin 1c	82.5	10.57	1.17e‐03	3.20e‐02
SKTI versus control (up)	TRINITY_DN183_c0_g1_i1	A0A7E5W745	carboxypeptidase B‐like	26218.4	9.59	2.84e‐09	4.97e‐07
SKTI versus control (up)	TRINITY_DN2479_c0_g1_i2	A0A2A4J4T7	Peptidase_M14 domain‐containing protein	1812.8	9.06	1.56e‐14	1.70e−11
SKTI versus control (up)	TRINITY_DN229_c2_g1_i1	A0A2A4JFE4	Peptidase S1 domain‐containing protein	39.8	8.57	1.70e‐05	1.00e‐03
SKTI versus control (up)	TRINITY_DN1830_c0_g1_i2	A0A7E5WD72	Methionine aminopeptidase	21.4	8.06	3.99e‐04	1.38e‐02
SKTI versus control (up)	TRINITY_DN5782_c0_g2_i2	A0A2W1C1V2	Presequence protease, mitochondrial	322.8	7.82	1.02e‐05	6.42e‐04
SKTI versus control (up)	TRINITY_DN3216_c0_g1_i7	A0A2W1BFJ3	Peptidase S1 domain‐containing protein	88.2	7.56	5.79e‐04	1.86e‐02
SKTI versus control (up)	TRINITY_DN773_c0_g1_i3	A0A7E5WY52	trypsin, alkaline C‐like isoform X1	57.7	5.91	7.39e‐04	2.24e‐02
SKTI versus control (up)	TRINITY_DN4381_c0_g1_i1	A0A7E5WEG8	trypsin, alkaline C‐like	2318.6	5.17	1.24e‐05	7.56e‐04
SKTI versus control (up)	TRINITY_DN1704_c1_g1_i1	A0A2A4K0B4	Peptidase S1 domain‐containing protein	18467.4	5.11	7.16e−12	3.00e‐09
SKTI versus control (up)	TRINITY_DN773_c0_g1_i8	A0A7E5WEG8	trypsin, alkaline C‐like	3391.9	5.04	1.98e‐04	7.88e‐03
SKTI versus control (up)	TRINITY_DN756_c3_g2_i3	A0A7E5WEG8	trypsin, alkaline C‐like	215.6	3.33	8.29e‐04	2.45e‐02
SKTI versus control (up)	TRINITY_DN473_c0_g1_i2	B4X992	Chymotrypsin‐like protease C9	2825.2	3.13	2.11e‐03	4.95e‐02
SKTI versus control (up)	TRINITY_DN1205_c0_g3_i1	C9W8H3	Serine protease 11	10834.5	3.11	1.11e‐03	3.07e‐02
SKTI versus control (up)	TRINITY_DN1688_c0_g1_i1	A0A7E5WEG8	trypsin, alkaline C‐like	12799.1	2.98	6.57e‐04	2.04e‐02
SKTI versus control (up)	TRINITY_DN2644_c0_g1_i7	A0A2A4J379	Peptidase_M14 domain‐containing protein	765.7	2.90	7.52e‐05	3.53e‐03
SKTI versus control (up)	TRINITY_DN514_c0_g2_i5	A0A6J2K2L8	Aminopeptidase	26646.9	2.23	3.67e‐06	2.55e‐04
SKTI versus control (up)	TRINITY_DN2119_c0_g1_i2	A0A2W1BRL8	Peptidase S1 domain‐containing protein	153.3	1.94	9.25e‐04	2.66e‐02
SKTI versus control (up)	TRINITY_DN1671_c0_g1_i8	Q95WL1	Aminopeptidase	32558.9	1.73	1.33e‐03	3.51e‐02
SKTI versus control (up)	TRINITY_DN3365_c0_g1_i4	A0A5J6YEK3	Aminopeptidase N 1	350.8	1.63	1.32e‐03	3.50e‐02
SKTI versus control (up)	TRINITY_DN438_c0_g1_i3	A0A286LPZ7	Aminopeptidase	35117.3	1.46	1.45e‐03	3.76e‐02
SKTI versus control (up)	TRINITY_DN1726_c0_g1_i3	A0A2A4J912	Signal peptidase complex subunit 2	2157.2	1.01	1.24e‐04	5.43e‐03
SKTI versus control (down)	TRINITY_DN2549_c0_g2_i6	A0A7E5VVD6	Aminopeptidase	108.8	−1.39	9.27e‐05	4.21e‐03
SKTI versus control (down)	TRINITY_DN1349_c3_g1_i1	A0A7I0ZHR5	pyroglutamyl‐peptidase 1	6543.3	‐2.95	9.97e‐04	2.82e‐02
SKTI versus control (down)	TRINITY_DN9357_c0_g1_i1	A0A7E5V9F9	cysteine proteinase 15A‐like	82.2	‐7.22	8.34e‐04	2.46e‐02
SKTI versus control (down)	TRINITY_DN2257_c0_g1_i3	D2Y4U5	Trypsin	13.8	‐7.76	6.30e‐08	6.57e‐06
SKTI versus control (down)	TRINITY_DN5915_c0_g1_i8	A0A7E5VH23	dipeptidyl aminopeptidase‐like protein 6	21.1	‐8.37	2.34e‐09	4.21e‐07
SKTI versus control (down)	TRINITY_DN11_c0_g1_i2	A0A2H1V4U2	Kynureninase	22.2	‐21.22	5.71e‐08	6.06e‐06
SKTI versus control (down)	TRINITY_DN8796_c0_g1_i8	A0A7E5W3M5	CLIP domain‐containing serine protease	39.8	−22.66	6.69e‐09	1.03e‐06
SKTI versus control (down)	TRINITY_DN61_c0_g2_i12	A0A7E5WM93	chymotrypsin‐2‐like	4934.1	−29.95	1.77e‐14	1.82e‐11
GORE‐2 versus control (up)	TRINITY_DN2479_c0_g1_i3	A0A2A4J4T7	Peptidase_M14 domain‐containing protein	399.3	24.87	3.82e‐11	1.19e‐08
GORE‐2 versus control (up)	TRINITY_DN1362_c1_g1_i14	A0A6J1WCK9	cysteine proteinase B‐like	278.2	11.47	1.01e‐03	2.69e‐02
GORE‐2 versus control (up)	TRINITY_DN1830_c0_g1_i2	A0A7E5WD72	Methionine aminopeptidase	21.4	7.28	1.39e‐03	3.41e‐02
GORE‐2 versus control (up)	TRINITY_DN183_c0_g1_i1	A0A7E5W745	carboxypeptidase B‐like	26218.4	7.26	6.88e‐06	4.57e‐04
GORE‐2 versus control (up)	TRINITY_DN2479_c0_g1_i2	A0A2A4J4T7	Peptidase_M14 domain‐containing protein	1812.8	7.20	1.04e‐09	2.09e‐07
GORE‐2 versus control (up)	TRINITY_DN6754_c1_g2_i2	A0A7E5VCF3	sentrin‐specific protease 8 isoform X1	9.6	7.17	1.14e‐03	2.95e‐02
GORE‐2 versus control (up)	TRINITY_DN5782_c0_g2_i2	A0A2W1C1V2	Presequence protease, mitochondrial	322.8	6.08	6.06e‐04	1.85e‐02
GORE‐2 versus control (up)	TRINITY_DN773_c0_g1_i5	A0A7E5WY52	trypsin, alkaline C‐like isoform X1	3299.6	3.96	1.72e‐03	3.95e‐02
GORE‐2 versus control (up)	TRINITY_DN1688_c0_g2_i1	A0A7E5WEG8	trypsin, alkaline C‐like	780.7	2.98	7.89e‐05	3.81e‐03
GORE‐2 versus control (up)	TRINITY_DN6055_c0_g1_i3	A0A2A4K2D7	Peptidase S1 domain‐containing protein	107.7	2.34	2.00e‐03	4.41e‐02
GORE‐2 versus control (up)	TRINITY_DN3365_c0_g1_i4	A0A5J6YEK3	Aminopeptidase N 1	350.8	2.30	5.67e‐06	3.86e‐04
GORE‐2 versus control (up)	TRINITY_DN1671_c0_g1_i8	Q95WL1	Aminopeptidase	32558.9	2.04	1.50e‐04	6.42e‐03
GORE‐2 versus control (up)	TRINITY_DN2119_c0_g1_i2	A0A2W1BRL8	Peptidase S1 domain‐containing protein	153.3	1.92	1.03e‐03	2.73e‐02
GORE‐2 versus control (up)	TRINITY_DN1671_c0_g1_i3	Q95WL1	Aminopeptidase	61095.5	1.83	1.05e‐03	2.76e‐02
GORE‐2 versus control (up)	TRINITY_DN514_c0_g2_i5	A0A6J2K2L8	Aminopeptidase	26646.9	1.72	3.38e‐04	1.19e‐02
GORE‐2 versus control (up)	TRINITY_DN2549_c0_g2_i4	A0A7E5VVD6	Aminopeptidase	446.0	1.65	1.56e‐03	3.69e‐02
GORE‐2 versus control (up)	TRINITY_DN438_c0_g1_i3	A0A286LPZ7	Aminopeptidase	35117.3	1.41	2.14e‐03	4.64e‐02
GORE‐2 versus control (up)	TRINITY_DN569_c0_g1_i2	A0A7E5WZL7	Carboxypeptidase Q	3660.8	1.15	1.00e‐03	2.68e‐02
GORE‐2 versus control (up)	TRINITY_DN569_c0_g1_i6	A0A7E5WZL7	Carboxypeptidase Q	2866.0	1.02	4.68e‐05	2.46e‐03
GORE‐2 versus control (down)	TRINITY_DN1344_c0_g1_i2	A0A7E5WTN1	dipeptidyl peptidase 9 isoform X2	1520.4	−1.43	2.15e‐04	8.48e‐03
GORE‐2 versus control (down)	TRINITY_DN7975_c0_g1_i5	A0A8B8HUT4	probable alpha‐aspartyl dipeptidase isoform X1	220.6	−1.59	2.89e‐04	1.06e‐02
GORE‐2 versus control (down)	TRINITY_DN2549_c0_g2_i6	A0A7E5VVD6	Aminopeptidase	108.8	−1.72	1.97e‐06	1.50e‐04
GORE‐2 versus control (down)	TRINITY_DN468_c0_g1_i1	A0A7E5WWZ6	cathepsin L‐like	45856.9	−1.82	1.36e‐03	3.35e‐02
GORE‐2 versus control (down)	TRINITY_DN15016_c0_g1_i1	A0A7E5W3X7	CLIP domain‐containing serine protease	20.0	−2.23	8.96e‐04	2.47e‐02
GORE‐2 versus control (down)	TRINITY_DN18996_c0_g1_i1	K4Q332	Caspase‐1	1259.6	−2.61	5.74e‐04	1.78e‐02
GORE‐2 versus control (down)	TRINITY_DN1752_c0_g1_i3	A0A2A4JJA9	Peptidase S1 domain‐containing protein	871.2	−2.81	5.59e‐06	3.82e‐04
GORE‐2 versus control (down)	TRINITY_DN9357_c0_g1_i6	A0A7E5V9F9	cysteine proteinase 15A‐like	1601.4	−3.37	4.13e‐05	2.22e‐03
GORE‐2 versus control (down)	TRINITY_DN10435_c0_g1_i1	O18442	Trypsin‐like protease	191.4	−5.65	3.85e‐04	1.31e‐02
GORE‐2 versus control (down)	TRINITY_DN2257_c0_g1_i3	D2Y4U5	Trypsin	13.8	−7.57	1.29e‐07	1.23e‐05
GORE‐2 versus control (down)	TRINITY_DN5915_c0_g1_i8	A0A7E5VH23	dipeptidyl aminopeptidase‐like protein 6	21.1	−8.19	5.20e‐09	8.02e‐07
GORE‐2 versus control (down)	TRINITY_DN3388_c0_g1_i1	A0A7E5VCR2	matrix metalloproteinase‐14 isoform X3	23.0	−8.24	3.99e‐04	1.34e‐02
GORE‐2 versus control (down)	TRINITY_DN5289_c0_g1_i4	A0A7E5V9F0	probable isoaspartyl peptidase/L‐asparaginase GA20639 isoform X2	68.9	−9.16	6.17e‐11	1.76e‐08
GORE‐2 versus control (down)	TRINITY_DN3388_c0_g1_i11	A0A7E5VCR2	matrix metalloproteinase‐14 isoform X3	70.7	−9.65	5.62e‐11	1.65e‐08
GORE‐2 versus control (down)	TRINITY_DN7975_c0_g1_i6	A0A8B8HUT4	probable alpha‐aspartyl dipeptidase isoform X1	205.9	−9.80	1.92e‐12	9.16e‐10
GORE‐2 versus control (down)	TRINITY_DN61_c0_g2_i11	Q3ZJD2	Midgut chymotrypsin	1068.6	−10.46	1.81e‐03	4.10e‐02
GORE‐2 versus control (down)	TRINITY_DN3388_c0_g1_i8	A0A7E5VCR2	matrix metalloproteinase‐14 isoform X3	156.0	−10.81	2.03e‐06	1.54e‐04
GORE‐2 versus control (down)	TRINITY_DN667_c0_g1_i4	A0A7E5VW75	putative serine protease K12H4.7	467.5	−10.94	9.43e‐05	4.44e‐03
GORE‐2 versus control (down)	TRINITY_DN9184_c0_g1_i11	A0A8B8IQW6	chymotrypsin‐2‐like	412.0	−12.47	1.41e‐03	3.43e‐02
GORE‐2 versus control (down)	TRINITY_DN9184_c0_g1_i2	A0A6J1P306	chymotrypsin‐2‐like	18411.9	−14.00	2.41e‐08	3.01e‐06
GORE‐2 versus control (down)	TRINITY_DN9184_c0_g1_i10	A0A2A4JX80	Peptidase S1 domain‐containing protein (Fragment)	38513.8	−14.13	2.36e‐09	4.03e‐07
GORE‐2 versus control (down)	TRINITY_DN61_c0_g2_i6	Q3ZJD2	Midgut chymotrypsin	7121.7	−15.38	6.55e‐06	4.40e‐04
GORE‐2 versus control (down)	TRINITY_DN9184_c0_g1_i9	A0A2A4JX80	Peptidase S1 domain‐containing protein (Fragment)	5569.5	−29.09	9.59e‐14	6.84e‐11
GORE‐2 versus control (down)	TRINITY_DN61_c0_g2_i12	A0A7E5WM93	chymotrypsin‐2‐like	4934.1	−29.75	2.61e‐14	2.12e‐11

*Note:* Seventy‐five differentially expressed protease calls are listed, corresponding to 60 unique transcripts; 15 transcripts responded to both inhibitors in the same direction. Log2 fold‐change values are unshrunken DESeq. 2 estimates.

**FIGURE 6:**
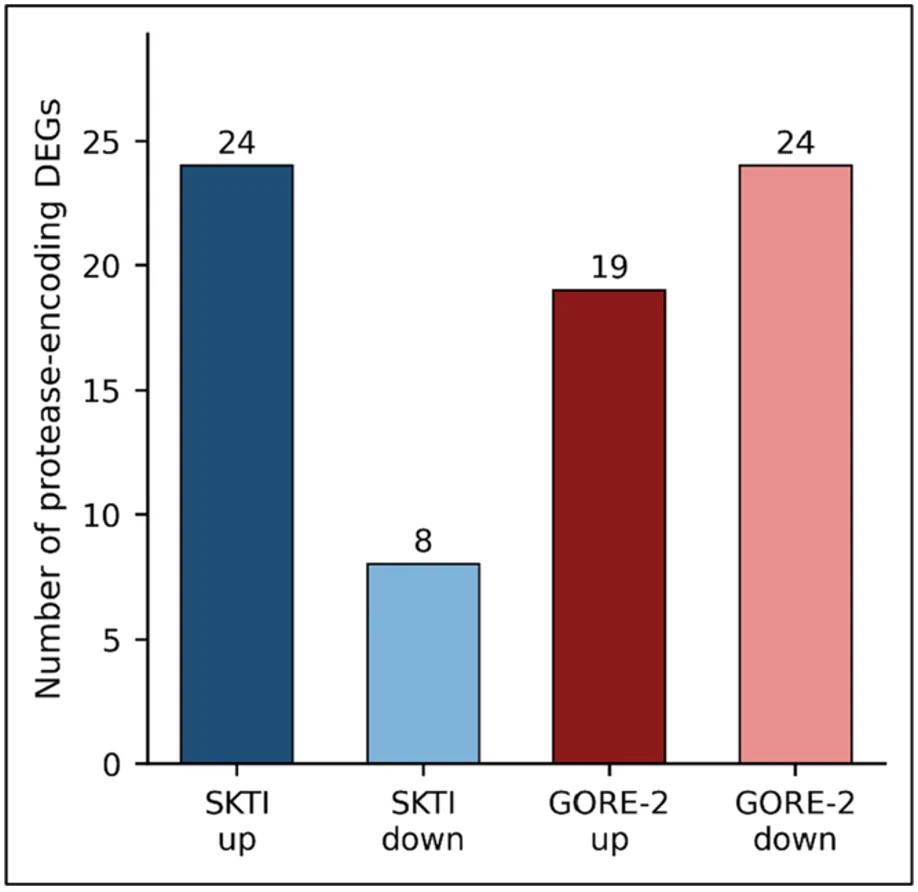
Number of genes encoding proteases that were differentially expressed in the midgut of *A. gemmatalis* larvae fed with SKTI and GORE‐2. SKTI: 24 up‐regulated and 8 down‐regulated; GORE‐2: 19 up‐regulated and 24 down‐regulated.

Transcriptomic profiling revealed DEGs encoding proteolytic enzymes (Table 1), highlighting the extensive modulation of digestive and regulatory proteases by both inhibitors. In larvae fed with SKTI, protease genes were up‐regulated, including multiple alkaline trypsin‐like and other S1‐family serine proteases, a chymotrypsin‐like protease C9, carboxypeptidase B‐like and M14‐family carboxypeptidases, aminopeptidases (including aminopeptidase N1), a methionine aminopeptidase and a mitochondrial presequence protease, while down‐regulated transcripts comprised a chymotrypsin‐2‐like protease, a CLIP‐domain serine protease, trypsin, cysteine proteinases and a dipeptidyl aminopeptidase.

In contrast, GORE‐2 treatment induced a distinct transcriptional signature. Among the up‐regulated genes, several encode serine proteases and dipeptidyl peptidases (DPP3 and DPP9), as well as ATP‐dependent zinc metalloproteases (YME1) and collagenase‐like proteins, suggesting activation of proteolytic systems associated with mitochondrial and cellular stress responses. Down‐regulated transcripts were enriched for aminopeptidases N, metalloproteases (NAS‐4‐like, zinc carboxypeptidases), and chymotrypsin‐1, indicating suppression of major digestive enzymes in the midgut (Figure 6).

Notably, exclusive dysregulation patterns were observed for each inhibitor: 13 protease genes were uniquely upregulated by SKTI and 8 by GORE‐2, reflecting PI‐specific modulation of proteolytic pathways. These results suggest that SKTI primarily induces a compensatory upregulation of serine proteases, whereas GORE‐2 exerts broader repression of zinc‐ and aminopeptidase genes, potentially interfering with larval digestion and proteostasis mechanisms.

### 3.6 Metatranscriptomic Analysis

Taxonomic and functional profiling of the *Anticarsia gemmatalis* midgut transcriptome generated a comprehensive overview of the 42,372 de novo–assembled unigenes, annotated using the TrapID pipeline and eggNOG‐mapper v2 (Figure 7). At the phylum level, the vast majority of annotated sequences (~85%) were assigned to Arthropoda, confirming the predominance of host‐derived transcripts. A smaller proportion corresponded to Bacteria (7%), Ascomycota (2%), and other minor lineages (6%), consistent with a low but detectable presence of microbial and fungal sequences likely associated with the gut microbiota or environmental contamination.

**FIGURE 7:**
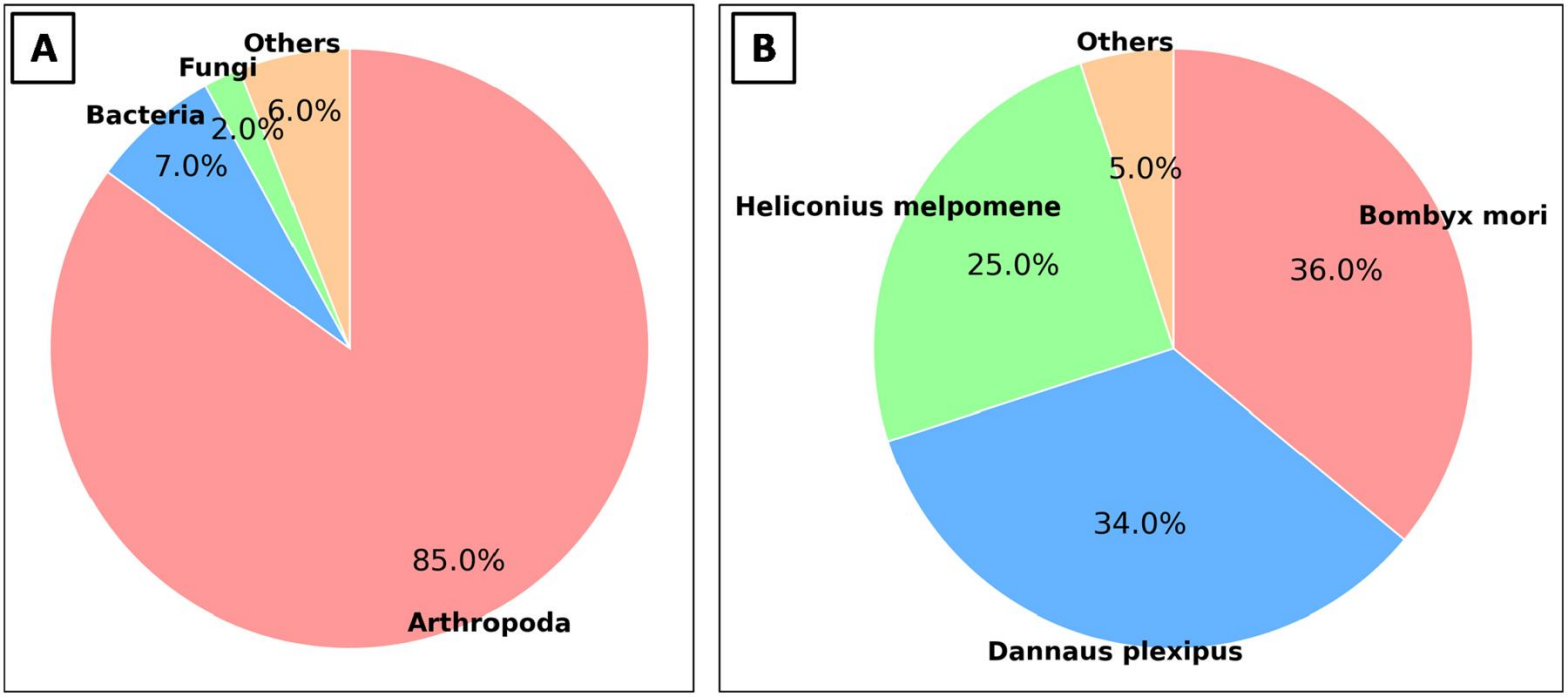
Comprehensive taxonomic, functional, and length‐based characterization of 42,372 de novo–assembled unigenes from *A. gemmatalis* processed through the TRAPID pipeline. All transcripts were annotated using eggNOG‐mapper v2. (A) Phylum‐level distribution shows that Arthropoda accounted for 85% of annotated hits, followed by Bacteria (7%), Ascomycota (2%), and other lineages (6%), indicating minor contributions from microbes and fungi. (B) Species‐level homology profiling against the eggNOG 5.0 database revealed best‐hit proportions to *Bombyx mori* (36%), *Danaus plexippus* (34%), *Heliconius melpomene* (25%), and other taxa (5%), reflecting strong similarity to available Lepidopteran reference proteomes.

At the species level, homology analysis revealed a strong affiliation of the transcriptome to available Lepidopteran reference proteomes, particularly *Bombyx mori* (36%), *Danaus plexippus* (34%), and *Heliconius melpomene* (25%), with the remaining 5% distributed among other insect taxa. This taxonomic pattern underscores the high‐quality assembly and the evolutionary proximity of *A. gemmatalis* to model Lepidoptera with well‐characterized genomes.

Functionally, the annotated unigenes exhibited broad representation across metabolic, structural, and regulatory categories, including genes involved in digestion, detoxification, redox homeostasis, and cuticular development, consistent with the physiological relevance of midgut tissues for nutrient assimilation and defense responses. The presence of bacterial and fungal orthologs in the dataset may reflect symbiotic or transient microbial communities that contribute to digestive and metabolic processes in the larval midgut.

The metatranscriptomic profiling of the *A. gemmatalis* midgut transcriptome revealed a complex and taxonomically diverse microbial community associated with the caterpillar digestive tract (Figure 8). At the domain level, most transcripts of non‐host origin were assigned to Bacteria, whereas Archaea and Viruses accounted for a minor fraction of the detected sequences (Figure 8A). Within the bacterial domain, the most represented orders were Burkholderiales, Chitinophagales, Micrococcales, and Clostridiales, taxa frequently reported in insect midguts for their involvement in nutrient recycling, xenobiotic degradation, and cell‐wall polysaccharide breakdown.

**FIGURE 8:**
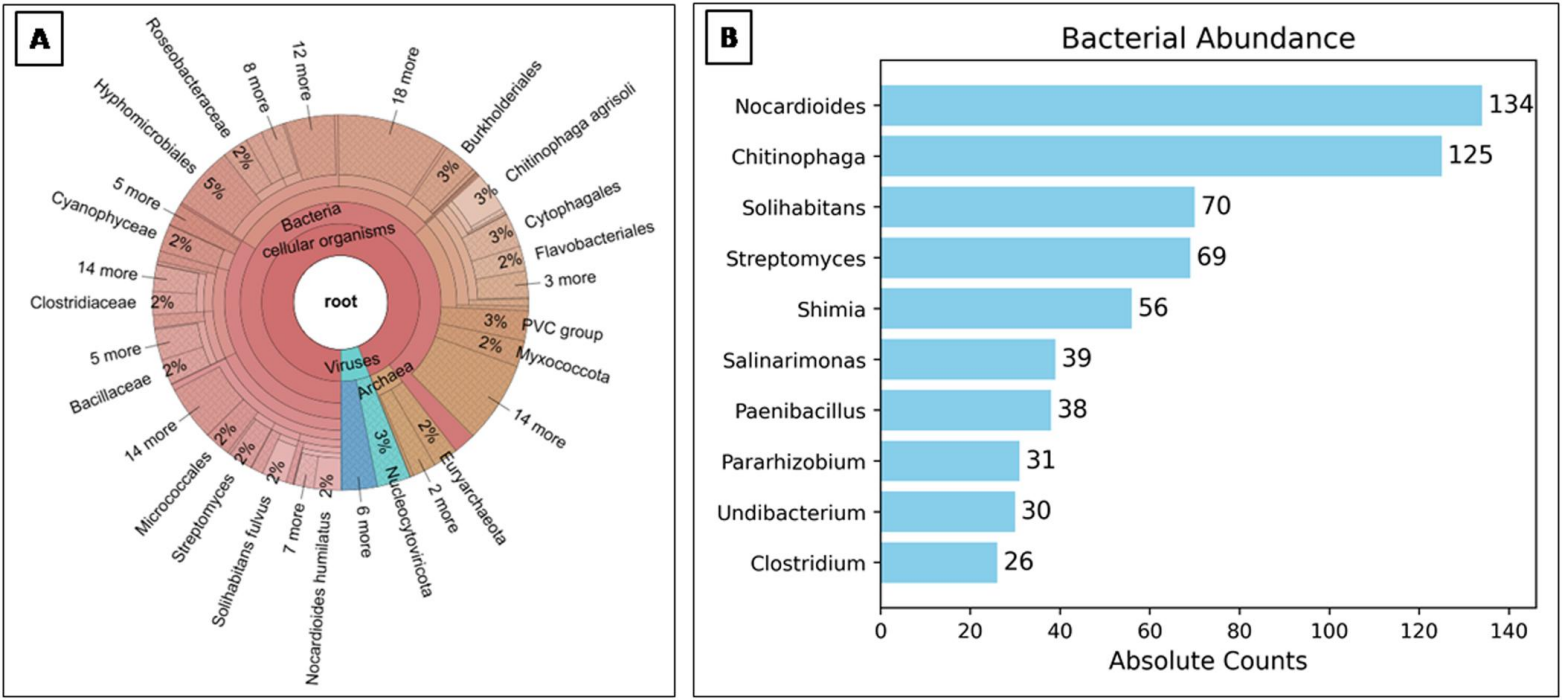
Taxonomic profile of microorganisms detected in the midgut of *Anticarsia gemmatalis* based on RNA‐Seq data. (A) Sunburst chart showing the taxonomic distribution of detected organisms, highlighting the domains *Bacteria*, *Archaea*, and *Viruses*. Most non‐host transcripts were assigned to *Bacteria*, predominantly from the orders *Burkholderiales*, *Chitinophagales*, *Micrococcales*, and *Clostridiales*. (B) Bar chart showing the absolute abundance of the main bacterial genera. *Nocardioides* and *Chitinophaga* were the most represented, followed by *Solihabitans* and *Streptomyces*, indicating a taxonomically diverse gut microbiome in the host insect.

At the genus level, Nocardioides and Chitinophaga showed the highest relative transcript abundance, followed by Solihabitans and Streptomyces (Figure 8B). Members of these genera are known for their metabolic versatility, including the capacity to produce hydrolytic enzymes, antimicrobial metabolites, and detoxifying oxidoreductases, suggesting potential contributions to host digestion and defense.

The presence of multiple bacterial lineages with diverse metabolic capabilities indicates that *A. gemmatalis* harbors a functionally heterogeneous gut microbiome, likely aiding nutrient assimilation, maintaining gut homeostasis, and protecting against dietary or environmental stressors.

### 3.7 Metatranscriptomic Insights Into the Functional Roles of Gut‐Associated Bacteria in *Anticarsia gemmatalis*

Functional annotation of bacterial transcripts from the *A. gemmatalis* midgut metatranscriptome revealed metabolically active microbial communities contributing to multiple core biochemical processes (Figure 9). KEGG pathway enrichment analysis (Figure 9A) indicated that most bacterial transcripts were associated with central carbon metabolism, including glycolysis, the tricarboxylic acid (TCA) cycle, pyruvate metabolism, and the pentose phosphate pathway. These findings suggest that gut‐associated bacteria participate in energy production, generation of reducing power, and intermediary metabolism, thereby complementing host nutrient assimilation and maintaining redox homeostasis. In addition, transcripts associated with amino acid biosynthesis, lipid metabolism, and cofactor and vitamin production were detected, indicating a biosynthetically versatile microbiota capable of supporting host metabolic demands.

**FIGURE 9:**
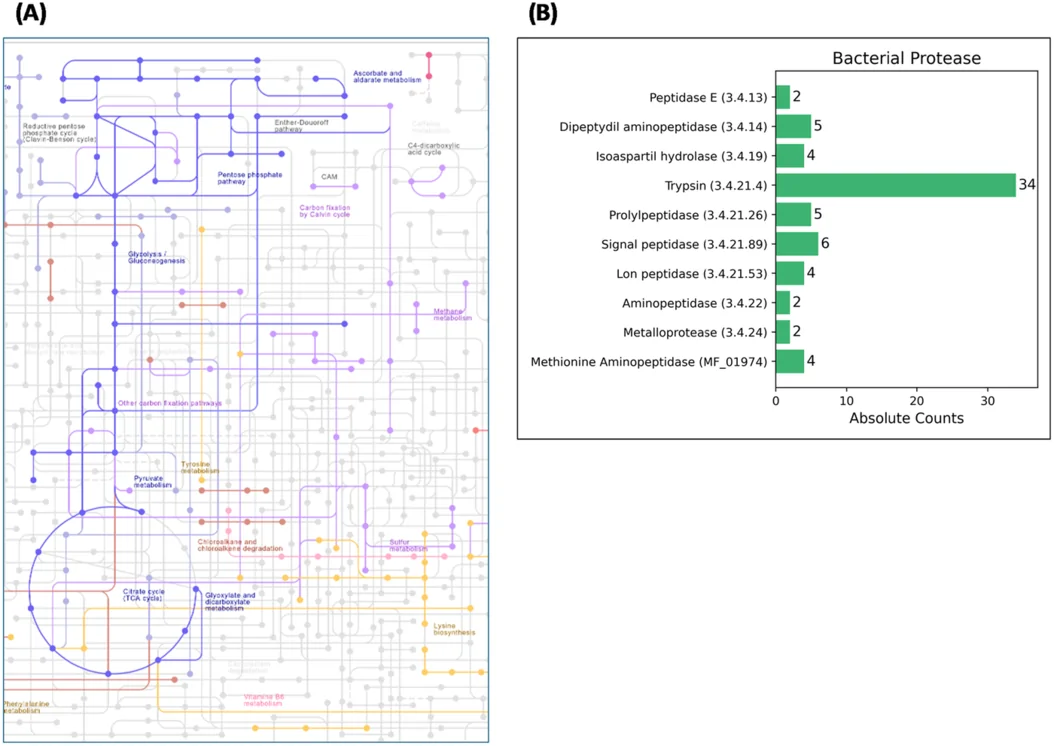
Functional analysis of bacterial transcripts detected in the midgut of *Anticarsia gemmatalis* based on RNA‐Seq data. (A) Enriched bacterial metabolic pathways associated with transcripts identified in the *A. gemmatalis* gut. Highlighted pathways include glycolysis, the tricarboxylic acid (TCA) cycle, pyruvate metabolism, and the pentose phosphate pathway, suggesting metabolically active and diverse bacterial communities. (B) Absolute abundance of bacterial proteases annotated from gut metatranscriptomic data. Bacterial trypsin (EC 3.4.21.4) was the most represented protease, followed by signal peptidase (EC 3.4.21.89) and other peptidase families, indicating a potential role of bacteria in digestion and proteolytic modulation within the insect gut. Collectively, bacterial proteases accounted for approximately 7% of all microbial transcripts, underscoring their functional relevance within the *A. gemmatalis* intestinal microbiome.

Analysis of bacterial proteolytic enzymes (Figure 9B) revealed that trypsin‐like serine proteases (EC 3.4.21.4) were the most abundant, followed by signal peptidases (EC 3.4.21.89) and other peptidase families. Collectively, bacterial proteases accounted for approximately 7% of total microbial transcripts, underscoring their functional significance in gut digestion and proteolytic modulation. Thus, the functional profile of microbial transcripts suggests that the *A. gemmatalis* gut microbial community is transcriptionally and enzymatically active, supporting a cooperative network of nutrient processing, detoxification, and digestive regulation that may influence the insect's adaptation to plant‐derived protease inhibitors.

Comparative transcriptomic analysis detected no significant differential expression of bacterial genes in response to SKTI or GORE‐2 (Figures 8 and 9). However, because bacterial transcripts accounted for only a minor fraction of the whole‐midgut RNA‐Seq data, these results should be interpreted with caution. Under the sequencing depth and analytical framework used here, the bacterial fraction appeared transcriptionally stable across treatments.

## 4 Discussion

The present results demonstrate that the physiological effects of protease inhibitors extend beyond digestive inhibition and involve integrated alterations in midgut homeostasis. Although both SKTI and GORE‐2 modulated digestive and antioxidant pathways, the response patterns differed markedly between treatments. SKTI induced a compensatory response characterized by increased digestive proteases and antioxidant activation, whereas GORE‐2 promoted broader repression of digestive enzymes, weaker antioxidant responses, and more severe epithelial disruption. Together, these findings indicate that GORE‐2 imposes a stronger physiological burden on the larval midgut by simultaneously impairing digestive and protective functions.

The predominance of trypsin‐like transcripts reinforces the central role of serine proteases in lepidopteran digestion and highlights these enzymes as strategic molecular targets for peptide‐based pest control. The high representation of detoxification‐associated enzymes, including glucuronosyltransferases and cytochrome P450 monooxygenases, further supports the metabolic plasticity of *A. gemmatalis* in response to dietary stressors and xenobiotics.

Although GORE‐2 induced broader repression of digestive proteases, SKTI promoted the up‐regulation of multiple protease genes, consistent with the adaptive response commonly observed in herbivorous insects exposed to plant‐derived protease inhibitors. Generalist lepidopterans frequently compensate for PI exposure by overexpressing or diversifying digestive proteases, including inhibitor‐insensitive isoforms, as previously reported for Helicoverpa armigera exposed to SKTI (Singh et al. 2020; Hafeez et al. 2021; Chamani et al. 2025; Zhang et al. 2024).

Beyond its predicted interaction with trypsin‐like enzymes, GORE‐2 induced broader transcriptional suppression of digestive proteases associated with protein processing and nutrient assimilation. This broader suppression likely reflects a disruption of digestive homeostasis in the midgut caterpillars (Hafeez et al. 2021). Because these enzymes act cooperatively in protein digestion and nutrient assimilation, their simultaneous repression may compromise intestinal metabolic balance and increase physiological stress. Such digestive impairment may also contribute to the accumulation of reactive oxygen species (ROS), since nutritional imbalance and epithelial dysfunction are closely associated with mitochondrial stress and oxidative disturbance in insect midgut tissues (Felton and Summers 1995; Jaffar et al. 2022; Sies 1991).

Both SKTI and GORE‐2 induced antioxidant responses characterized by increased CAT, SOD, GST, and POX activities, indicating activation of redox‐regulatory and detoxification pathways under PI exposure. However, antioxidant induction was consistently weaker under GORE‐2 treatment, suggesting reduced physiological compensation against oxidative stress. In insects, increased antioxidant activity is commonly associated with adaptation to xenobiotics and dietary stressors, whereas insufficient activation may favor ROS accumulation and cellular injury (Farahani et al. 2020; Jaffar et al. 2022; Felton and Summers 1995). The more robust antioxidant response observed under SKTI exposure likely indicates the coevolutionary adaptation of *A. gemmatalis* to soybean‐derived inhibitors (Zhu‐Salzman and Zeng 2015). Meanwhile, the diminished enzymatic activation caused by GORE‐2 underscores the potential of synthetic peptide inhibitors to interfere with compensatory digestive and protective mechanisms in insect pests.

The weaker activation of antioxidant enzymes in GORE‐2‐treated caterpillars was accompanied by severe epithelial injury, including extensive vacuolization and disruption of the striated border. This association suggests that antioxidant compensation was insufficient to offset the oxidative stress induced by digestive disruption. In insects, CAT, SOD, GST, and POX act cooperatively to neutralize superoxide radicals, hydrogen peroxide, and lipid peroxidation products (Jaffar et al. 2022; Felton and Summers 1995; Birben et al. 2012). Therefore, the comparatively reduced induction of these enzymes under GORE‐2 exposure may have favored ROS accumulation, membrane destabilization, and progressive epithelial deterioration. The histological alterations observed here are consistent with oxidative damage patterns previously reported in Lepidoptera exposed to insecticides or Bt toxins, including cytoplasmic disorganization, epithelial detachment, and loss of intestinal barrier integrity (Fiaz et al. 2018; Paulo, Meriño‐Cabrera, et al. 2026).

These findings collectively underscore the potential of non‐plant protease inhibitors, such as GORE‐2, to interfere with the midgut's digestive and protective functions in insect pests. In the current study, GORE‐2 was associated with a broader suppression of digestive proteases, diminished antioxidant activation, and markedly higher histological lesion scores compared with SKTI. This response pattern aligns with a reduced capacity for physiological compensation and/or increased cytotoxic stress. Although the specific roles of impaired physiological compensation and direct cellular toxicity remain indistinguishable within this study, these results substantiate the promise of synthetic peptide inhibitors as viable candidates for integrated pest management strategies (de Almeida Barros et al. 2021; Paulo, Schultz, et al. 2026; Singh et al. 2020; de Andrade et al. 2026).

Because all analyses were performed at a standardized 24 h exposure point, the present study provides an integrated view of the early sublethal response to PI ingestion. Within this experimental window, the observed patterns reflect the combined transcriptional, biochemical, and histological adjustments triggered in the larval midgut, rather than discrete sequential stages of response. This temporal framework is also relevant when interpreting host‐microbiome associations, which may involve subtler or later shifts than those captured at this stage (Dong et al. 2025).

Metatranscriptomic analyses revealed a taxonomically diverse set of putative gut‐associated microbial transcripts linked to metabolic, proteolytic, and detoxification‐related functions in the larval midgut. However, because no treatment‐associated differential expression was detected in the bacterial fraction and the microbial signal was derived from whole‐midgut RNA‐seq data, these findings should be interpreted as exploratory and do not demonstrate a functional contribution of the microbiota to the response to PI exposure. Collectively, the observed transcriptomic, biochemical, and histological responses indicate that GORE‐2 exposure was associated with broader disruption of digestive protease expression, distinct antioxidant responses, and epithelial alterations in the larval midgut. These patterns are consistent with altered digestive homeostasis, although the underlying causal mechanisms remain to be experimentally established.

## 5 Conclusions

This study demonstrates that *A. gemmatalis* larvae respond to SKTI and GORE‐2 through clearly distinct midgut molecular and physiological patterns. SKTI induced a compensatory response characterized by the up‐regulation of digestive proteases, antioxidant enzymes, and detoxification‐related pathways, consistent with adaptation to a plant‐derived inhibitor. In contrast, GORE‐2 was associated with broader repression of digestive proteases, weaker activation of antioxidant responses, and more pronounced epithelial disruption, indicating a greater intestinal stress burden under the conditions tested. Although the midgut microbiome was taxonomically diverse and functionally associated with metabolic and proteolytic processes, no treatment‐associated differential expression was detected in the bacterial fraction within the present analytical framework. Overall, the results identify host digestive, redox, and histological responses as the primary treatment‐sensitive components and support GORE‐2 as a promising peptide scaffold for future studies to develop selective insect control strategies. However, additional investigations addressing peptide stability, target selectivity, non‐target safety, and performance under field‐relevant conditions will be necessary before practical application can be considered.

## Author Contributions

**Neilier Rodrigues da Silva Júnior:** conceptualization; investigation; project administration; writing – original draft. **Eulálio Gutemberg Dias dos Santos:** conceptualization; investigation; methodology; software; resources; data curation. **Daniel Guimarães Silva Paulo:** Conceptualization; Writing – review and editing; Formal analysis; Data curation. **Yaremis Meriño‐Cabrera:** Writing – review and editing; Visualization; Validation. **Ian de Paula Alves Pinto:** Methodology; Formal analysis. **Maria Julia Summy:** Conceptualization; Visualization. **Ian Lucas Batista Santos:** Visualization. **Milena Godoi Lima:** Visualization. **Maria Clara Neves Gomes Rodrigues:** Visualization. **Rafael Junior de Andrade:** Visualization. **José Eduardo Serrão:** Methodology; Visualization; Investigation. **Elizabeth Pacheco Batista Fontes:** Visualization; Supervision. **Humberto Josué Oliveira Ramos:** Supervision; Formal analysis; Funding acquisition. **Maria Goreti de Almeida Oliveira:** Funding acquisition; Supervision; Formal analysis.

## Ethics Statement

The work did not involve ethical questions and all authors agreed to participate in this research study.

## Consent

All authors are in accordance with the publication of the manuscript.

## Conflicts of Interest

The authors declare no conflicts of interest.

## Supporting information


Supporting File

## Data Availability

RNA‐seq data and transcriptome generated in this study were deposited into the NCBI Sequence Read Archive (BioProject: PRJNA1494060).
