# Supplementary material for: Integrated Transcriptomic and Physiological Analyses Reveal Disruption of Digestive Homeostasis Induced by Protease Inhibitors in Anticarsia gemmatalis Caterpillars
Source: Arch Insect Biochem Physiol. 2026 Oct 4;123(2):e70223. doi: 10.1002/arch.70223 (PMC13634601; doi:10.1002/arch.70223)
Supplement: Supplementary file 1 [file ARCH-123-e70223-s001.docx]

**Table S1**: *A. gemmatalis* reference assembly statistics

**Score:**

Total trinity 'genes': 20,192

Total trinity transcripts: 51,825

Percent GC: 40.41

**Statistics based on all transcripts:**

Contig N50: 3,246

Median contig length: 1,617

Average contig: 2,243.60

Total assembled bases: 116,274,451

**Statistics based on longest isoform per ‘’gene’’:**

Contig N50: 2,891

Median contig length: 1,204

Average contig: 1906,23

Total assembled bases: 38,490,674
